# Zika Virus Impairs Neurogenesis and Synaptogenesis Pathways in Human Neural Stem Cells and Neurons

**DOI:** 10.3389/fncel.2019.00064

**Published:** 2019-03-15

**Authors:** Livia Rosa-Fernandes, Fernanda Rodrigues Cugola, Fabiele Baldino Russo, Rebeca Kawahara, Caio Cesar de Melo Freire, Paulo Emílio Corrêa Leite, Ana Carolina Bassi Stern, Claudia Blanes Angeli, Danielle Bruna Leal de Oliveira, Stella Rezende Melo, Paolo Marinho de Andrade Zanotto, Edison Luiz Durigon, Martin Røssel Larsen, Patricia Cristina Baleeiro Beltrão-Braga, Giuseppe Palmisano

**Affiliations:** ^1^Department of Biochemistry and Molecular Biology, University of Southern Denmark, Odense, Denmark; ^2^Department of Microbiology, Institute of Biomedical Science, University of São Paulo, São Paulo, Brazil; ^3^Department of Parasitology, Institute of Biomedical Science, University of São Paulo, São Paulo, Brazil; ^4^Department of Genetics and Evolution, Federal University of São Carlos, São Carlos, Brazil; ^5^Institute of Biophysics Carlos Chagas Filho, Federal University of Rio de Janeiro, Rio de Janeiro, Brazil; ^6^School of Arts Sciences and Humanities, University of São Paulo, São Paulo, Brazil

**Keywords:** Zika virus, microcephaly, congenital Zika syndrome, neurogenesis, synaptogenesis

## Abstract

Growing evidences have associated Zika virus (ZIKV) infection with congenital malformations, including microcephaly. Nonetheless, signaling mechanisms that promote the disease outcome are far from being understood, affecting the development of suitable therapeutics. In this study, we applied shotgun mass spectrometry (MS)-based proteomics combined with cell biology approaches to characterize altered molecular pathways on human neuroprogenitor cells (NPC) and neurons derived from induced pluripotent stem cells infected by ZIKV-BR strain, obtained from the 2015 Brazilian outbreak. Furthermore, ZIKV-BR infected NPCs showed unique alteration of pathways involved in neurological diseases, cell death, survival and embryonic development compared to ZIKV-AF, showing a human adaptation of the Brazilian viral strain. Besides, infected neurons differentiated from NPC presented an impairment of neurogenesis and synaptogenesis processes. Taken together, these data explain that CNS developmental arrest observed in Congenital Zika Syndrome is beyond neuronal cell death.

## Introduction

Zika Virus (ZIKV) is a flavivirus first isolated in non-human primate in 1947 in the ZIKA Forest in Uganda (Dick et al., 1952). Even though mosquitos from the species Aedes are considered the main transmitting vectors, sexual and transplacental forms of transmission have been described (Besnard et al., 2014; D’Ortenzio et al., 2016; Turmel et al., 2016; Russo and Beltrao-Braga, 2017; Russo et al., 2017). From 1960’s until recent past, ZIKV infection were described to be either asymptomatic or associated with conjunctivitis, fever, maculopapular rash, and arthralgia (Duffy et al., 2009). However, recently, ZIKV infection has been associated with a congenital syndrome in newborns called Congenital Zika Syndrome (CZS) (Cugola et al., 2016; Franca et al., 2016), which is characterized by a congenital malformation spectrum including microcephaly, intracranial calcifications, eye abnormalities or other congenital central nervous system-related abnormalities (Franca et al., 2016; Kindhauser et al., 2016; Alvarado and Schwartz, 2017). Although pulmonary and musculoskeletal disorders have been reported, ZIKV effect on nervous system is the most pronounced characteristic of the Brazilian outspread, characterizing a new circulating strain called ZIKV-BR (Cugola et al., 2016; Franca et al., 2016). Indeed, neural progenitor cells (NPC) seem to be the main affected population by ZIKV with consequent cell death (Cugola et al., 2016; Chavali et al., 2017), impairment of cell cycle progression (Tang et al., 2016), premature differentiation and defective cell division (Gabriel et al., 2017) leading to depletion of neural progenitors of the cortical layer.

Despite the recent massive efforts to understand ZIKV effect on neuronal biology, pathways involved in the physiopathology of the disease promoted by different strains in diverse individuals are still poorly understood, limiting the development of therapeutic options. Comprehensive omics methodologies can assist on deciphering complex networks and especially how they can interact to affect disease outcome.

Here, we applied shotgun mass spectrometry-based proteomics combined with microscopy and cell biology approaches to characterize altered molecular pathways on NPC infected by ZIKV-BR strain. The effect of most studied African ZIKV strain MR766 (ZIKV-AF) on NPC was also evaluated. A direct comparison between ZIKV-BR and ZIKV-AF infection of NPC revealed strain-specific regulated pathways and phenotypes. Furthermore, analysis of stem cells differentiated neurons exhibited impairment of neurogenesis and synaptogenesis upon ZIKV infection, revealing that mechanisms under CNS malformations were beyond cell death. Taken together, our results highlight the effects of ZIKV infection in the CNS under development, as a dynamic and comprehensive molecular mechanism for ZIKV-BR brain malformation.

## Materials and Methods

### Subjects Ascertainment

Subjects were recruited through The Tooth Fairy Project (University of São Paulo – USP), after approval by the Ethics Committee of the Institute of Biosciences CEP-ICB/USP (Protocol CEP/ICB-USP 1001). After a complete description of the study, parents provided a written informed consent. The cell lineage used in this study was derived from a male Caucasian health donor and has been previously described (Cugola et al., 2016; Mesci et al., 2018; Russo et al., 2018).

### Cell Culture

Stem Cells from Human Exfoliated Deciduous Teeth (SHED) were isolated, characterized and reprogramed as previously reported (Beltrao-Braga et al., 2011). Induced Pluripotent Stem Cells (iPSC) colonies were plated on matrigel (BD Biosciences) coated plates and kept for 5 days with mTSeR media (Stem Cell Technologies). On the fifth day, the media was changed to N2 media (DMEM/F12 media supplemented with 1× N2 supplement (Invitrogen) and dual SMAD inhibitors 1 μM of dorsomorphin (Tocris) and 10 μM of SB431542 (Stemgent), for 48 h. After 2 days in this condition, colonies were removed from the plate and cultured in suspension as Embryoid Bodies (EBs) for 5 days at 90 rpm in N2 media with the dual SMAD inhibitors. EBs were plated on matrigel plates with NGF media composed of: DMEM/F12 media supplemented with 0.5× N2, 0.5× Gem21 supplement (Gemini Bio-products), 20 ηg/mL of FGF2 and 1% penicillin/streptomycin. Emerged rosettes were manually picked, dissociated and plated on polyOn and Laminin (10 μg/mL poly-ornithine and 2.5 μg/mL laminin). NPCs population obtained was expanded using NGF media. G-banding karyotype was performed in all NPCs by Children’s Hospital of Los Angeles (Los Angeles, CA, United States). Abnormal karyotypes cell lines were discarded. To differentiate NPCs into neurons, a confluent plate was treated with 10 μM of ROCK inhibitor for 48 h (Y-27632, Calbiochem) in the absence of FGF, with regular media changes every 4 days, for a total of 28 days. All cultures were incubated at 37°C in 5% CO_2_ high-humidity environment.

### *In vitro* ZIKV Infection

Viral preparations used here were described previously (Cugola et al., 2016). NPCs and neurons were infected with 1 MOI of ZIKV-BR, ZIKV-AF, and MOCK (control without virus). For viral adsorption, cells in monolayer were incubated for 1 h at 4°C with gentle agitation every 10 min. Next, the inoculum was removed, and cells were washed once with PBS. Culture medium was added to each well, and cells were incubated at 37°C and 5% CO_2_. 24 h after infection, NPCs were scraped and kept under agitation to form 3D neurospheres (NS) and for the duration of the experiment. Measurements of the NPCs neurospheres diameter were performed in the infected and MOCK conditions. One-way ANOVA with Tukey’s multiple comparison test was used to identify the statistically significant comparisons. ^∗^Adjusted *p*-value ≤ than 0.05. ^∗∗∗^Adjusted *p*-value ≤ than 0,001. Ns, not significant with adjusted *p*-value > than 0.05.

In addition, in order to assess if ZIKV was able to cause neurogenesis or synaptogenesis impairment, NPCs in monolayer were infected with 1 MOI and differentiated into neurons for 28 days. For MOCK controls, the same volume of supernatant was added to each experiment, and the same procedures were followed.

### Real-Time PCR

RNA was extracted from supernatant of cells using TRIzol reagent, following manufacturer’s instructions (Invitrogen). All RNA pellet was resuspended in 30 μl of RNase-free distilled water, quantified using a NanoDrop spectrophotometer (NanoDrop Technologies) and stored at -80°C. The set of primer/probe specific for ZIKV was synthesized by Sigma Life Science, with 5- FAM as the reporter dye for the probe. The set of primers/probe ZIKV 835, ZIKV 911c, and ZIKV 860-FAM were previously described (Lanciotti et al., 2008). The real-time reaction was performed with 10 μL of each sample and 10 μL of the AgPath-IDTM One-Step RT-PCR reagents (Applied Biosystems). The amplification was done in an Applied Biosystems 7500 real-time PCR system, and involved activation at 45°C for 15 min, 95°C for 15 min followed by 40 amplification cycles of 95°C for 15 s, 60°C for 15 s, and 72°C for 30 s. The real-time data were analyzed using SDS software from Applied Biosystems.

### Sample Preparation for Mass-Spectrometry Based-Proteomics

#### Protein Enzymatic Digestion and Chemical Labeling

Cell pellets were resuspended in denaturating buffer containing 6 M urea, 2 M thiourea, 50 mM triethylammonium bicarbonate (TEAB) and protease and phosphatase inhibitors. Extracted proteins were reduced at a final concentration of 10 mM DTT for 30 min at room temperature and subsequently alkylated in 40 mM iodoacetamide (IAA) for 30 min in the dark. The solution was further incubated at room temperature for 3 h after addition of Endoproteinase Lys-C (10 μg). Subsequently the urea concentration was diluted to less than 1 M and protein digestion was completed with the addition of trypsin 2% (w/w) overnight at room temperature. Samples were acidified to 1% formic acid and centrifuged at 14,000 × *g* for 10 min to stop trypsin digestion and remove insoluble material (e.g., lipids). Supernatant was collected and dried prior to desalting on an POROS Oligo R3 reversed-phase microcolumn as previously described (Palmisano et al., 2010). Protein and peptide quantification was performed using Qubit^TM^ fluorometric quantitation (Thermo Fisher Scientific). A total of 50 μg of peptides from each urine sample were diluted to a final concentration of 100 mM of TEAB and labeled with TMT10plex labeling reagent (Thermo Fisher Scientific) according to the manufacturer’s instructions. After the labeled peptides were combined, 50 μg aliquot was desalted on an POROS Oligo R3 reversed-phase microcolumn and eluted peptides were dried by vacuum centrifugation to produce the total peptide fraction.

#### Hydrophilic Interaction Liquid Chromatography (HILIC) Fractionation

Total peptide was reconstituted to 90% ACN, 0.1% TFA and injected onto an in-house packed TSKgel Amide-80 HILIC (Tosoh, 5 μm) 320 μm × 170 mm μHPLC column using an Agilent 1200 HPLC system (Palmisano et al., 2012). Both samples were eluted using a gradient from 90 to 60% ACN, 0.1% TFA over 42 min at a flow rate of 6 μl/min. Fractions were automatically collected at 1 min intervals after UV detection at 210 nm and the fractions were combined to a total of 11 fractions according to UV detection. All fractions were dried by vacuum centrifugation.

#### Reversed Phase Nano-Liquid Chromatography Tandem Mass Spectrometry (nLC-MS/MS)

Each HILIC fraction was resuspended in 0.1% FA, and peptides were loaded on an in-house packed pre-column (2 cm × 100 μm inner diameter 5 μm) using an Easy-nanoLC system (Thermo Fisher Scientific) and separated by gradient from 3 to 28% solvent B in 100 min, 28–45% in 20 min, 45–100% B in 5 min and 8 min at 100% B. (*A* = 0.1% FA; *B* = 90% ACN, 0.1% FA) at a flow of 250 nL/min on analytical Reprosil-Pur C18-AQ column (17 cm × 100 μm 3 μm inner diameter). The Easy-nanoLC system was connected online to a QExactive HF Hybrid Quadrupole-Orbitrap mass spectrometer (Thermo Fisher Scientific) operating in positive ion mode and using data-dependent acquisition. The Orbitrap acquired the full MS scan with an AGC target value of 3 × 10^6^ ions and a maximum fill time of 100 ms. Each MS scan was acquired at 120,000 resolution with a mass range of 400–1600 Da. The 10 most abundant peptide ions were selected for HCD fragmentation (collision energy: 29). Fragmentation was performed at 60,000 resolution for a target of 1 × 10^5^ and a maximum injection time of 200 ms using an isolation window of 1.2 m/z, dynamic exclusion of 30 s and fixed first mass 110 m/z.

### Database Searches and Bioinformatics Analyses

Raw data from large scale DDA proteomic experiments were processed using Proteome Discoverer v2.1.1.21 (Thermo Fisher Scientific) and searched against Uniprot human reference database (release April 2016, 20,201 sequences) including the genome polyprotein sequence from the MR766 ZIKV and common contaminants using both in-house Mascot server version 2.2.04 (Matrix Science Ltd.) and the embedded Sequest HT server. Database searches were performed with the following parameters: precursor mass tolerance of 10 ppm, product ion mass tolerance of 0.02 Da, allowing two missed cleavages for trypsin. Cysteine carbamidomethylation and TMT10plex labeling for lysine and N-terminal as fixed modification. Variable modifications were set as follow: Methionine oxidation, protein N-terminal acetylation, and asparagine deamidation. Percolator algorithm was used to select peptides with FDR < 1% and a *q*-value up to 0.01. Quantification was performed using the Proteome Discoverer workflow node “Reporter Ions quantifier” and performed on the log2-values of the measured normalized peptide abundances.

Three biological replicates were considered for the quantitative statistical analysis and all proteins identified with two or more peptides were processed using Perseus v1.5.3.2. Principal component analysis (PCA) was performed as exploratory data analysis approach in all identified proteins with two or more unique peptides. Quantitative analysis was carried out on the log2-values of the normalized abundances. Significantly regulated proteins of paired comparisons were evaluated by *t*-test analysis, while regulation among three conditions was accessed by ANOVA, both followed by Benjamini–Hochberg correction at 5% significance level (Benjamini and Hochberg, 1995). The term “up-regulated” is used to describe proteins and peptides more abundant in a given comparison, while “downregulated” refers to those less abundant ones. A specific database containing 253 microcephaly-associated proteins was extracted from the human Uniprot database.

Differentially regulated proteins were analyzed using Ingenuity Pathway Analysis software (IPA, Qiagen). Overrepresented pathways, upstream regulators and biological and cellular functions were derived from INGENUITY Pathway Analysis^1^. Protein-protein interaction networks were constructed using STRING software^2^ with High confidence parameter (0.700) uploaded in Cytoscape (version 3.5.1) followed by enrichment analysis with pug-in BINGO (version 3.0.3) with 5% FDR. Gene ontology, pathway and disease enrichment analysis were also carried out ToppFun function of toppGene suite^3^ with FDR correction of 1%.

### Parallel Reaction Monitoring Analysis

Parallel reaction monitoring was performed on Easy-nanoLC system connected online to a QExactive HF Hybrid Quadrupole-Orbitrap mass spectrometer (Thermo Fisher Scientific) operating in positive ion mode. Reversed phase chromatography gradient was from 3 to 28% solvent B in 52 min, 28–47% B in 5 min, 45–100% B in 5 min, and 8 min at 100% B. Full MS scan was acquired at 120,000 resolution, AGC target 3 × 106, maximum injection time of 100 ms and mass range 150–2000 Da. HCD fragmentation was performed using inclusion list with the following parameters: 60,000 for a target of 2 × 105 and a maximum injection time of 100 ms using an isolation window of 1.2 m/z and collision energy of 29, fixed first mass 110 m/z. The list of all peptides and m/z for PRM analysis are available at Supplementary Table S11.

### PRM-Like Data Analysis

Raw files were imported into MaxQuant version 1.5.2.8 and the database search engine Andromeda was used to search MS/MS spectra against a database composed of the Reviewed Uniprot Human Protein Database (release April 2016; 20,201 entries) with tolerance level of 4.5 ppm for MS and 20 ppm for MS/MS. Reporter ion MS2: 10plex TMT was included in the search parameters and enzyme specificity was set to trypsin with a maximum of two missed cleavages. Normalization was performed by dividing the reporter ion intensity of each target peptide by the sum of the respective report ion intensities from the two ACTB peptides. *T*-test analysis was performed between MOCK (*n* = 3) vs. ZA (*n* = 3) and MOCK (*n* = 3) vs. ZB (*n* = 3) and *p*-value < 0.05 was considered as statistically significant.

### Immunofluorescence

Cells were fixed using paraformaldehyde 4% in PBS (USB Corporation) for 15 min at room temperature. After washing, cells were permeabilized with 0.1% Triton X-100 (Promega) diluted in dPBS for 15 min. After blocking with 2% of BSA for 4 h, primary antibodies (described below) were incubated overnight at 4°C, and next day secondary antibodies (Life Technologies) were added and kept for 1 h at room temperature. Cells were washed and nucleus was stained with DAPI. Slides were mounted using Pro-long Gold antifade reagent (Invitrogen) (Supplementary Table S12).

### Neuronal Puncta Quantification

Neuroprogenitor cells were differentiated as previously described into neurons. After 4 weeks in culture, cells were fixed and stained for SYN1 (pre-synaptic protein) and Homer1 (post-synaptic protein) along with MAP2 (Microtubule-associated protein 2, a neuronal protein), for cell identification (Supplementary Table S12). Images were taken at 63× and synaptic puncta was characterized by pre-synaptic protein and post-synaptic protein colocalization. Puncta quantification was performed using considering number of puncta per neurite length. Five independent experiments were performed and *t*-test statistics was applied (^∗∗^*p*-value ≤ 0.01).

## Results and Discussion

### ZIKV-AF and ZIKV-BR Strains Induce Different Global Cellular Responses in Infected NPCs

**Figure 1:**
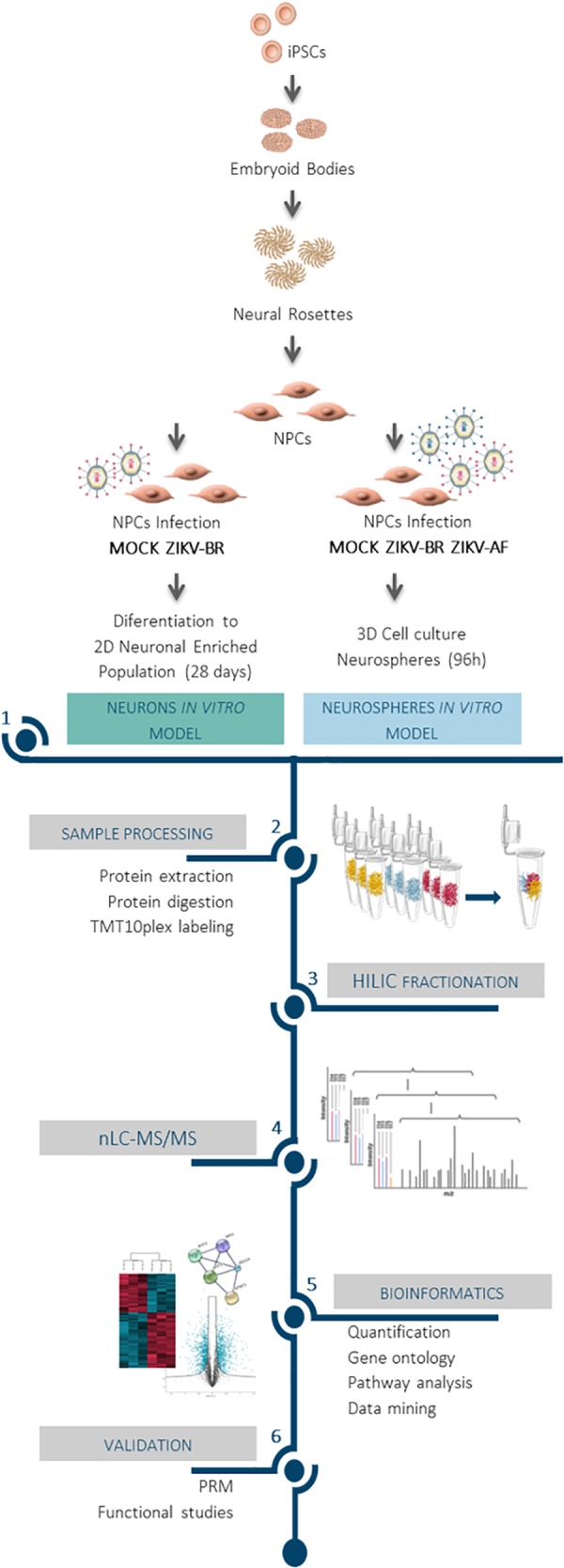
Impact of ZIKV infection on neuronal protein regulation revealed by mass spectrometry-based proteomics approach. Comprehensive proteomics workflow applied for the characterization of ZIKV infection protein regulation on neurodevelopment and host response. Human iPSC derived NPCs were infected and cultured as neurospheres (3D) for 96 h. In parallel, ZIKV-BR infected NPCs were differentiated into neurons (2D) for 28 days. After protein extraction, chemical labeling strategy (TMT10plex) was adopted, followed by HILIC fractionation and nLC-MS/MS analysis. This approach made possible the study of protein regulation upon viral infection to distinguish molecular signatures of ZIKV-AF and ZIKV-BR infection, assessing the host influence on protein regulation and the effect of ZIKV-BR on neurodevelopment *in vitro*.

**Figure 2:**
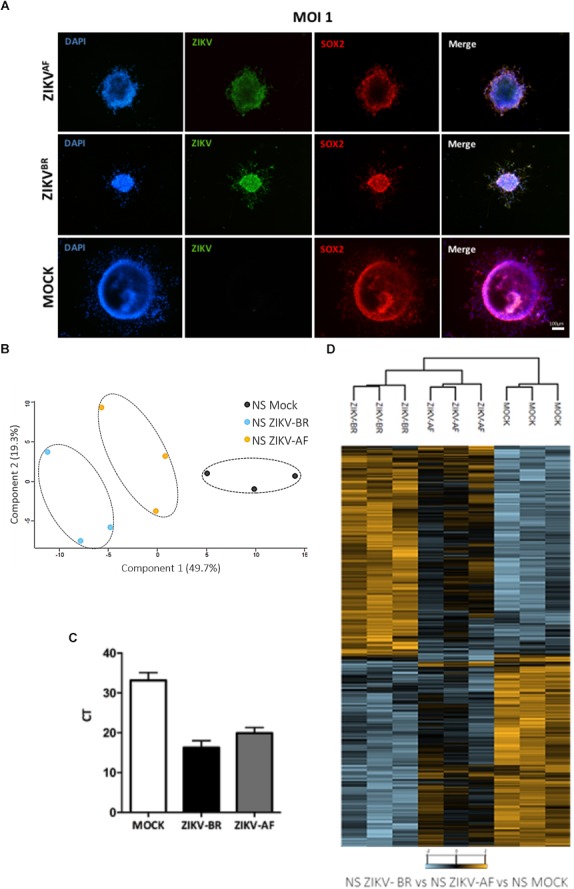
**(A)** Representative immunofluorescence microscopy of MOCK and ZIKV-BR infected neurospheres. DAPI (blue), ZIKV (green), and SOX2 (red). **(B)** Principal component analysis of all proteins quantified from human neurospheres reveals different protein regulation among viral strains. **(C)** Viral load detected by RT-qPCR. **(D)** Hierarchical clustering of regulated proteins after 96 h exposure to ZIKV-BR, ZIKV-AF, or cell alone (MOCK) (FDR < 5%).

To identify altered molecular pathways affected in CNS cells upon ZIKV infection, we generated human neural progenitor cells (NPCs) and neurons from iPSCs and infected these with ZIKV-BR and the *MR-766 strain* (ZIKV-AF) (Figure 1). NPCs were cultured as 3D-neurospheres (NS) and a comprehensive MS-based quantitative proteomics approach was applied after 96 h of ZIKV-AF, ZIKV-BR (1 MOI), or vehicle (MOCK) exposure (Figure 2A and Supplementary Figure S1A). A total of 6080 proteins were identified and 4579 proteins were quantified with two or more peptides based on TMT reporter ion intensity before statistical analysis. All quantified proteins were first explored by principal component analysis (PCA) that displayed three distinct clusters according to their abundance variation (Figure 2B). Relative quantification comparison showed 935 differentially regulated proteins among NS MOCK, NS ZIKV-BR, and NS ZIKV-AF (FDR < 5%) (Supplementary Table S1). These changes are independent of the infection rate, which was similar between the two strains (Figure 2C). Unsupervised hierarchical clustering analysis of regulated proteins showed distinct protein profile between NS MOCK and NS ZIKV-BR, while NS ZIKV-AF presented a discrete profile with minor protein variation (Figure 2D and Supplementary Table S1). Indeed, ZIKV-AF did not have any protein significantly regulated compared to MOCK. ZIKV-AF was the first isolated strain (Dick et al., 1952) and initially used to test the virus ability to infect NPCs and immature neurons *in vitro* (Cugola et al., 2016; Tang et al., 2016; Gabriel et al., 2017). Regardless of its ability to induce cell growth arrest, molecular responses caused by ZIKV-AF contrast from those ZIKV obtained from more recent outbreaks (Gabriel et al., 2017). As a matter of fact, before more recent outbreaks reported in Pacific Islands and Americas (Duffy et al., 2009; Besnard et al., 2014; Franca et al., 2016), ZIKV African isolates were described as a mild infection without association to congenital malformations (Kindhauser et al., 2016).

A ZIKV phylogenetic study might shed some light into evolution of the disease. As previously reported, strains belonging to the Asian lineage have been associated to CZS (Kindhauser et al., 2016). Besides, one study associated the microcephaly with a single mutation in ZIKV Asian strain (Yuan et al., 2017).

**Figure 3:**
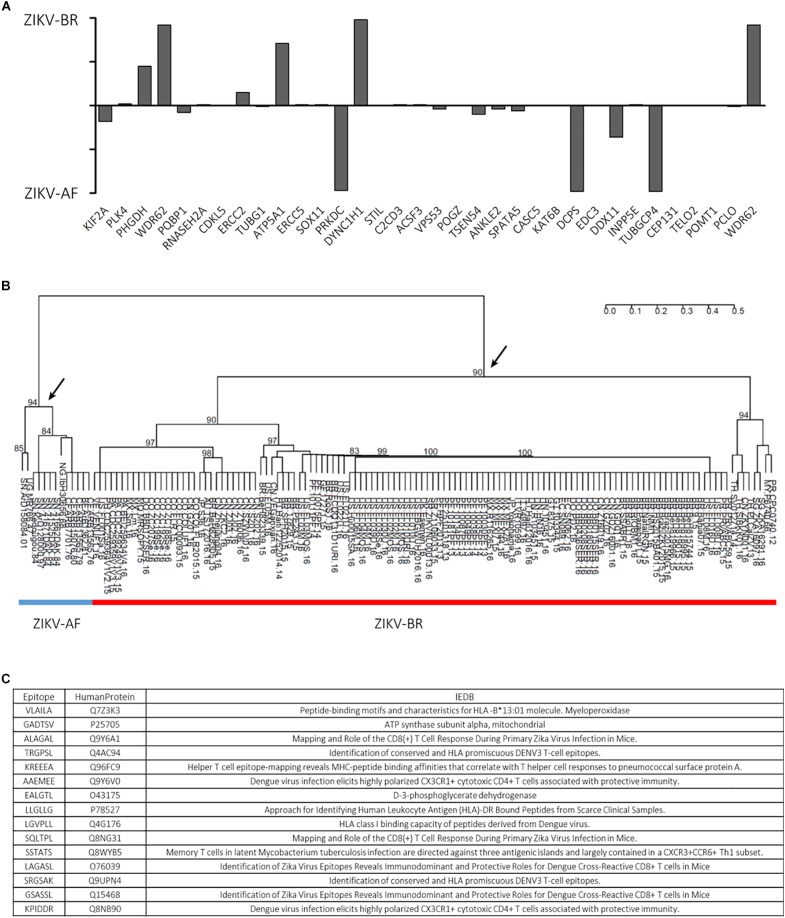
**(A)** ZIKV-BR and ZIKV-AF lineages have different epitopes matching human microcephaly-related proteins. **(B)** ZIKV-AF (blue line) and ZIKV-BR (red line) phylogenetic profile based on hexameric oligopeptides sequences shared with human microcephaly proteins. **(C)** List of selected epitopes and respective human proteins shows that part of the shared epitopes has been assigned to human proteins associated to DENV immune response according to IEDB database (http://www.iedb.org/).

Furthermore, an autoimmune etiological component for the CZS phenotype was suggested, as anti-viral immune response could potentially cross-react with human proteins based on common peptides with ZIKV-AF polyprotein (Lucchese and Kanduc, 2016). Since cross-reactivity following pathogen infection could generate autoimmune response, *in silico* generated oligopeptides shared between 136 ZIKV strains polyproteins and human proteins associated with microcephaly were evaluated. A total of 13 African lineages shared 341 oligopeptides while 126 Asian lineages shared 3243 oligopeptides with human proteins involved in microcephaly. Specific oligopeptides were associated to the different ZIKV strains, highlighting a strain-specific response (Figure 3A). Moreover, the hierarchical clustering analyses revealed that the shared hexameric sequences were informative to discriminate the major ZIKV lineages: Asian and African (the arrows in the figure indicate the nodes), pointing a phylogenetic structure in the shared oligopeptides between Zika lineages and human microcephaly proteins (Figure 3B). Interestingly, according to IEDB database^4^, part of shared epitopes has been assigned to human proteins associated to DENV immune response (Figure 3C). Since ZIKV-AF presented no significant statistical change in a pairwise comparison (NS ZIKV-AF vs. NS MOCK), we focused on the regulated pathways during ZIKV-BR infection in NS.

### ZIKV-BR Promotes Major Differential Protein Expression and Activation of Viral Transcription Machinery in Infected Neurospheres

**Figure 4:**
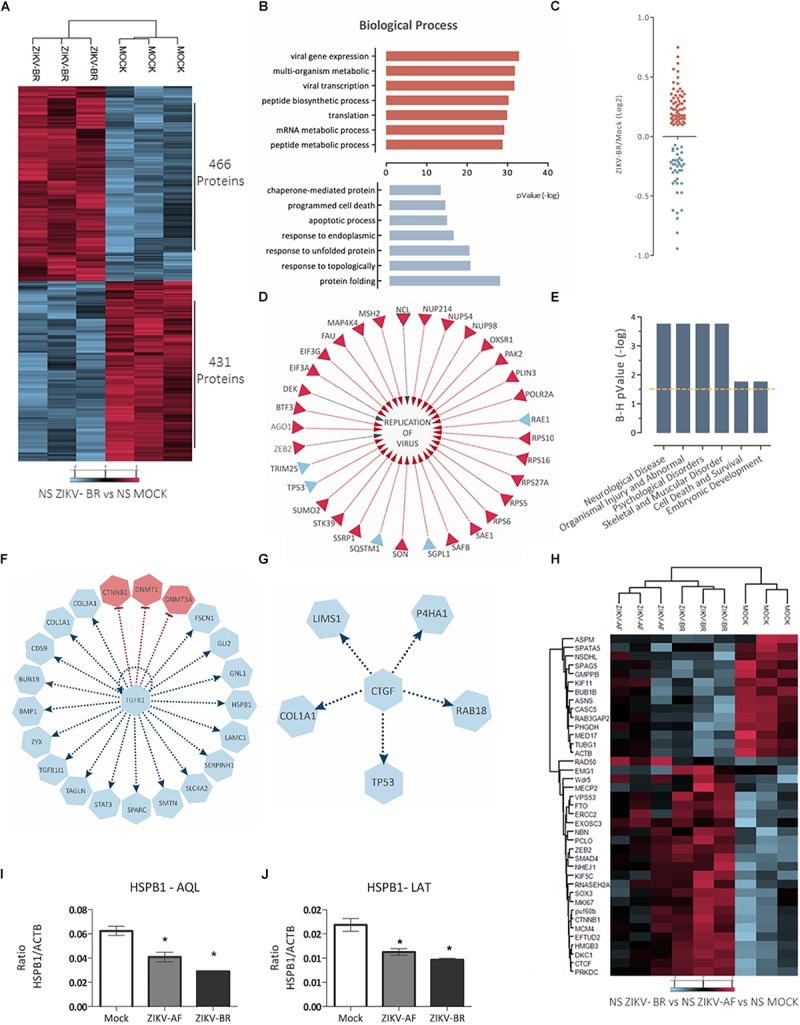
ZIKV-BR infection on neurospheres induces protein regulation in neurodevelopment and microcephaly associated pathways. **(A)** ZIKV-BR promotes changes in protein expression in neurospheres as shown by heatmap based on non-supervised hierarchical clustering. 466 proteins were found upregulated whereas 431 proteins where found downregulated in ZIKV-BR infected neurospheres in comparison to control condition (MOCK) (FDR < 5%). **(B)** Top enriched Gene Ontology Biological Process (GO) for proteins upregulated (red) and downregulated (blue) in ZIKV-BR infected neurospheres (*p* < 0.01). **(C)** Distribution of viral related protein expression in ZIKV-BR infected neurospheres. **(D)** Expression pattern of proteins involved in viral replication. **(E)** Predicted enriched diseases and functions in ZIKV-BR infected neurospheres (FDR < 5%). **(F)** Protein network involved in negative regulation of growth factor TGFβ1 and **(G)** Connective-tissue growth factor (CTGF), (upregulated proteins and processes are represented in red and downregulated in blue). **(H)** Hierarchical clustering of microcephaly related proteins (FDR < 5%) showing distinct expression patterns between MOCK and ZIKV-BR and discrete alterations after ZIKV-AF exposure. **(I,J)** Parallel reaction monitoring confirmed downregulation of HSPB1 (*p* < 0.001). LATQSNEITIPVTFESR and AQLGGPEAAK peptides belonging to HSPB1 protein were monitored for their abundance in the MOCK, ZIKV-AF, and ZIKV-BR conditions.

Focusing on the effect of ZIKV-BR on NPCs neurospheres, comparison between ZIKV and MOCK-infected cells showed 897 regulated proteins, being 466 up-regulated and 431 downregulated (Figure 4A and Supplementary Table S2). Distribution of regulated protein expression according to its function or localization showed mitochondrial, histones and ribosomal proteins were up-regulated, while heat shock proteins were downregulated (Supplementary Figure S2). Gene Ontology analysis showed “protein folding” as the most enriched Biological Process among downregulated proteins while “viral gene expression” and “viral transcription” were among most enriched Biological Processes of up-regulated proteins (Figure 4B and Supplementary Tables S3, S4), even though protein associated with viral process and positive regulation of virus replication could be found both up- and downregulated (Figure 4C,D). Disease and Bio Function analysis of all differentially regulated proteins upon ZIKV-BR infection showed Neurological Disease, Skeletal, and Muscular Disorder and Embryonic Development among enriched annotations (FDR < 5%) (Figure 4E).

Prediction analysis also revealed proteins that may present a key role in CSZ. TGFB1 growth factor (Figure 4F), identified here as inhibited upstream regulator, is present in the neocortical proliferative zones and neurons in the cortical plate (Miller, 2003) and regulates different biological pathways in many cell types, including cell proliferation and migration (Miller, 2003). In the developing brain, TGFB1 has a role in neuronal migration during cortical lamination and it is responsible for maintaining neuronal integrity (Rodriguez-Martinez and Velasco, 2012). Mice with reduced TGFB1 expression display degenerative neurological process marked by increased number of apoptotic neurons and reduced neocortical presynaptic integrity (Brionne et al., 2003). In addition, reduced expression of laminin, a matricellular protein associated with neuronal differentiation and growth (Luckenbill-Edds, 1997), is a common feature between Tgfb1^-/-^ mice and ZIKV-BR infected neurospheres (0.74 ZIKV-BR/MOCK ratio) (Supplementary Table S2) contributing to a neurodegenerative phenotype (Brionne et al., 2003).

Connective-tissue growth factor (CTGF), a matricellular protein involved in chondrogenesis and wound healing, was predicted as an inhibited upstream regulator (Figure 4G; Roberts et al., 1986). CTGF works as a downstream mediator of TGFB1 (Song et al., 2007) being able to bind directly to TGFβ1 (Abreu et al., 2002). This interplay has been described in mesenchymal stem cell proliferation (Tarr et al., 2015) and CTGF ablation cause aberrations in bone formation and craniofacial birth defects in mice (Lambi et al., 2012; Tarr et al., 2015). Moreover, proteins previously related to microcephaly were found regulated in ZIKV-BR infected NS (Figure 4H and Supplementary Table S5).

### ZIKV-BR Infection Promotes Downregulation of Heat Shock Proteins in Neurospheres

HSPB1 was the most downregulated protein observed in infected NPCs (0.34 ZIKV-BR/MOCK ratio) (Supplementary Table S2), which was further confirmed by PRM analysis (Figure 4I,J). Downregulation of HSPB1 has been previously observed in the study of proteins involved in neuronal differentiation (Cheng et al., 2016). Moreover, silencing of HSPB1 promoted differentiation into neurons possessing the characteristics of functional glutamatergic neurons (Williams et al., 2006). HSPB1 downregulation during progression of neurodegenerative diseases resulted in a decrease of neuritic length and complexity in adult neurons and was associated to neuronal cell death (Williams et al., 2006; Thongtan et al., 2012). Different flavivirus have explored HSPs in their relation to the host cell. HSP70 and 90 have been described as possible receptor for Japanese encephalitis virus cellular entry (Thongtan et al., 2012) and to function as a chaperone in phases of Dengue virus cycle (Taguwa et al., 2015). Besides, HSPs response exerts a cytoprotective role by regulating the course of neuronal cell differentiation, neuritic growth, and axonal polarity (Williams et al., 2006; Yang et al., 2008; Benitez et al., 2014; Cheng et al., 2016). ZIKV-BR infected NS presented a global downregulation of HSPs (from 0.34 to 0.85 ZIKV-BR/MOCK ratio) (Supplementary Table S2), which could reduce cell protection against programmed cell death and promote premature and flawed neuronal precursor differentiation.

### Neuronal Cells Derived From NPCs Displayed Altered Protein Regulation After ZIKV-BR Infection

To model the effects of ZIKV-BR infection on neurogenesis, NPCs were infected with ZIKV-BR (MOI 1) and induced to neuronal differentiation for a total of 28 days (Figure 1 and Supplementary Figure S3A), after which, viral RNA was detected by RT-qPCR (Supplementary Figure S3B). Using nLC-MS/MS, we could identify 7317 proteins from which 5870 were quantified with two or more peptides. During neurogenesis, the number of apoptotic cells (cleaved caspase 3) was increased in cell cultures infected with ZIKV-BR when compared to MOCK (Supplementary Figure S3C).

**Figure 5:**
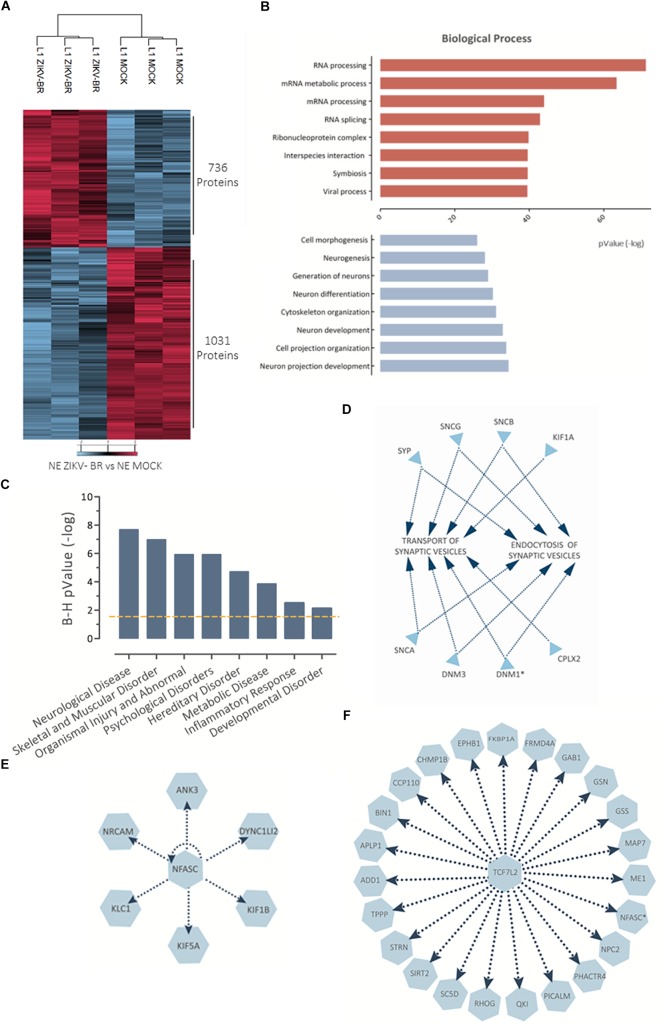
NPC-differentiated neurons show altered protein regulation after ZIKV-BR infection. **(A)** ZIKV-BR is able to promote greater protein regulation responses in neurons as shown by heatmap based on non-supervised hierarchical clustering (FDR < 5%). **(B)** Top enriched Gene Ontology Biological Process (GO) for proteins upregulated (red) and downregulated (blue) in ZIKV-BR infected neurons (*p* < 0.01). **(C)** Top enriched disease and functions for differentially regulated proteins in ZIKV-BR infected neurons (*p* < 0.05). **(D)** Enrichment analysis of proteins involved in synaptic vesicles transport and endocytosis according to IPA database. **(E)** Protein network involved in negative regulation of NFASC and **(F)** growth factor TCF7L2 (downregulated proteins and processes are represented in blue).

Comparison between ZIKV and MOCK-infected cells revealed 736 downregulated and 1031 upregulated proteins (Figure 5A and Supplementary Table S6). In a similar manner to observed in NS, ZIKV-BR infected neurons showed viral process enriched among upregulated proteins while neurogenesis, neural differentiation and neuron projection development were among the most enriched biological process on downregulated proteins (Figure 5B and Supplementary Tables S7, S8). Neurological impairment and skeletal and muscular disorder were the predicted diseases associated to all regulated proteins, as observed in CZS (Figure 5C,D).

Neurofascin, a cell adhesion molecule associated with induction of neurite outgrowth, among different roles in neural development (Burkarth et al., 2007) is another protein identified as downregulated in ZIKV-BR infected neurons (0.65 ZIKV-BR/MOCK) (Figure 5E and Supplementary Table S6). Another protein expressed in proliferating and differentiating neural stem cells is TCF7L2 (Korinek et al., 1998), which was predicted as inhibited transcription regulator in our model (Figure 5F). Thalamus axons elongation defects were observed in the absence of TCF7L2 associated with downregulation of guidance receptors NRP2, ROBO1, and ROBO2 (Lee et al., 2017), which were found downregulated in ZIKV-BR infected neurons (0.88, 0.70, and 0.77 ZIKV-BR/MOCK, respectively) (Figure 5F and Supplementary Table S6). TCF7L2 and NFASC network could be associated with dysfunctional neuron cells observed in CZS.

### Infection of NPCs With ZIKV-BR Leads to Synaptic Impairment in Differentiated Neurons

**Figure 6:**
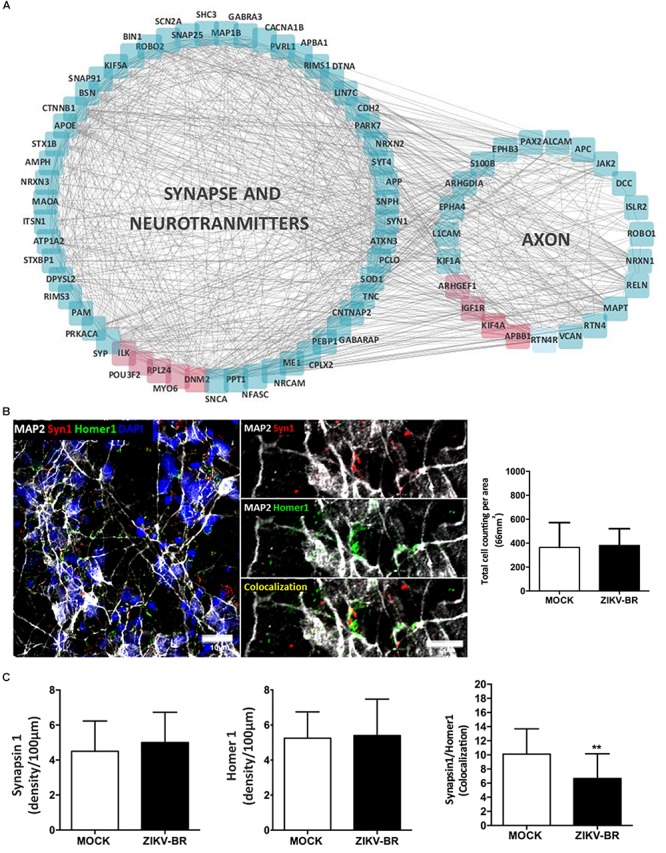
ZIKV-BR infection impairs synaptic transmission and neurodevelopment. **(A)** Protein network involved in synapse, neurotransmitters and axon related GO terms (downregulated proteins are displayed in blue and up-regulated in red). **(B)** Representative image of a co-localized puncta. **(C)** No difference in the number of pre- and post-synaptic proteins in ZIKV-BR infected neurons was observed. Decrease in co-localized SYN1 and Homer1 puncta in ZIKV-BR infected neurons was observed, with the same number of cells in culture. Data are represented as mean ± SEM, *t*-test statistics was applied. ^∗∗^*p*-value ≤ 0.01.

Synapse/neurotransmitters and axon were enriched among GO terms, indicating neuronal functional decrease after ZIKV-BR infection, with global downregulation of synaptic proteins (Figure 6A and Supplementary Tables S6, S9). Moreover, ZIKV-BR infected neurons featured downregulation of proteins associated with presynaptic precursor complex assembly and neurotransmitter release, such as VAMP2 and complexin 2, along with components of the presynaptic active zone, such as Piccolo, Bassoon and the SNARE proteins syntaxin and SNAP-25 (Waites et al., 2013; Mishima et al., 2014; Trimbuch and Rosenmund, 2016). In addition, post-synaptic density protein SHANK2 was found downregulated (Li and Sheng, 2003; Rizo and Xu, 2015). Beside extensive downregulation of synaptic proteins, synaptic loss in ZIKV-BR infected neurons was confirmed by quantification of colocalized presynaptic (SYN1) and post-synaptic (Homer1) puncta (Figure 6B,C). Downregulation of cell adhesion proteins Cadherin-13 and neurexin add to pathways involved in decreased synapse density observed ZIKV-BR infected neurons. Previous RNAi-based screen designed to study molecular mechanism of synapse development showed cadherin-13 knockdown reduces synapse density *in vitro* (Paradis et al., 2007). In a similar manner, neurexin disruption was shown to reduce active zone protein content, more specific Bassoon and RIM (Quinn et al., 2017). Taken together, these data show that ZIKV-BR infection is able to disrupt both synaptic formation and axodendritic stability. Besides neuronal cell death, aberrant protein expression in the synaptic region accompanied by synaptic loss could contribute to mental retardation and motor disabilities associated with CZS.

### ZIKV-BR Infection Promotes Aberrant Pattern of Neurogenesis Progression

**Figure 7:**
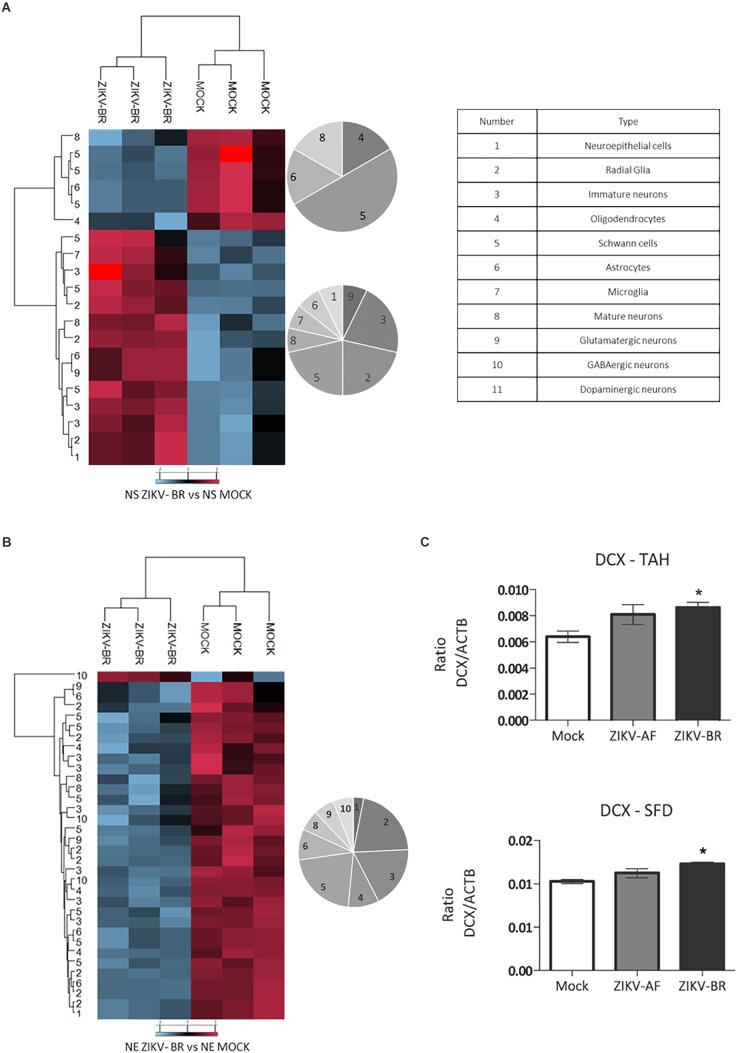
ZIKV-BR infection aberrant pattern of neurogenesis progression. Statistically regulated proteins between MOCK and ZIKV-BR infected NPCs and neurons were classified according to neuronal cell markers. The neural cell markers are for: 1. Neuroepithelial cells, 2. Radial glia, 3. Immature neurons, 4. Oligodendrocytes, 5. Schwann cells, 6. Astrocytes, 7. Microglia, 8. Mature neurons, 9. Glutamatergic neurons, 10. GABAergic neurons, and 11. Dopaminergic neurons. The neural cell markers are reported in Supplementary Table S10. **(A)** ZIKV-BR effect in neural cell markers in NPCs. **(B)** ZIKV-BR effect in neurons is shown by heatmap based on non-supervised hierarchical clustering of neuronal cell markers (FDR < 5%). **(C)** Parallel reaction monitoring confirmed upregulation of Doublecortin (DCX) in NPCs neurospheres (*p* < 0.001). TAHSFEQVLTDITEAIK and SFDALLADLTR peptides belonging to DCX protein were monitored for their abundance in the MOCK, ZIKV-AF, and ZIKV-BR conditions.

Embryonic neurogenesis is orchestrated by several cellular signaling events that influence the cell fate to the vast diversity of neuronal and glial cells that populate the mature brain. Markers belonging to 12 diverse neuronal cells were evaluated in ZIKV and MOCK infected NPCs and neurons (Supplementary Table S10). Cluster analysis revealed downregulation of Schwann cells and mature neurons and upregulation of radial glia and immature neurons markers in ZIKV-infected NS (Figure 7A,B and Supplementary Table S10). Doublecortin (DCX), a microtubule protein associated with migration process through the cortex expressed in young neurons (Gleeson et al., 1998, 1999; Francis et al., 1999), was found up-regulated in ZIKV-BR infected NS (1.75 ZIKV-BR/MOCK ratio) (Supplementary Table S2). DCX up-regulation was confirmed by PRM analysis of ZIKV-BR infected cells while no significant regulation was found in ZIKV-AF infected NS (Figure 7C). The analysis of neural markers expression in ZIKV-BR infected neurons showed a unique pattern with global downregulation of radial glia, immature neurons and Schwann cells (Figure 7B and Supplementary Table S10). These data agree with previous data that demonstrated premature differentiation in ZIKV-infected NPCs associated with neurogenesis impairment (Gabriel et al., 2017).

## Conclusion

The recent outbreak of ZIKV has attracted the attention of the public health systems due to the neurotropism of the virus that induces microcephaly, among other brain malformations in newborns. Understanding the molecular mechanisms of ZIKV induced microcephaly is a crucial step to identify potential therapeutic targets. As the molecular pathways behind the pathogenicity of ZIKV remain largely unknown, much effort has been made to characterize cellular responses to viral infection. While some studies have addressed the transcriptional changes upon ZIKV infection (Tang et al., 2016; Sanchez-San Martin et al., 2018), others have evaluated global protein regulation triggered by ZIKV infection of NPCs (Garcez et al., 2017; Jiang et al., 2018; Scaturro et al., 2018), revealing alterations of several pathways involved in cell cycle progression and cell death.

In the present study, the vulnerability of human NPCs and resulting neurons to ZIKV infection is explored using an unbiased large scale quantitative proteomic approach. Indeed, NPCs infected with ZIKV-AF and ZIKV-BR showed specific molecular pathways dysregulated leading to NPCs cell death and abnormal differentiation. A strain-specific protein modulation in NPCs was detected with ZIKV-BR inducing profound changes in NPCs neurospheres compared to ZIKV-AF. These data highlight the importance of viral adaptation and correlation with the disease. Moreover, the analysis of neurons differentiated after infection was applied as *in vitro* model of embryonic neurodevelopment upon ZIKV exposure. Infected mature neurons showed not only extensive downregulation of synapse-related proteins, but also decreased synapse density. This study broadens the understanding of protein expression changes in ZIKV-infected NPCs and neurons, revealing a strain-specific viral adaptation and functional impairment of surviving neurons.

## Data Availability

The datasets generated for this study can be found in ProteomeXchange Consortium via the PRIDE partner repository, PXD009293.

## Author Contributions

LR-F, PB-B, and GP did experimental design and wrote the manuscript. LR-F and GP did sample preparation and data analysis. FC and FR performed cell culture, viral infection, qPCR IF assays and synaptogenesis analysis. RK performed PRM data analysis. CdMF and PZ performed phylogenetic analysis. PL, AS, and CA assisted on sample preparation. DdO, SM, and ED performed viral isolation and titration. ML, PB-B, and GP supervised the project and wrote the manuscript. All authors revised the manuscript.

## Conflict of Interest Statement

The authors declare that the research was conducted in the absence of any commercial or financial relationships that could be construed as a potential conflict of interest.

## Footnotes

1 http://www.ingenuity.com

2 string-db.org

3 https://toppgene.cchmc.org/

4 http://www.iedb.org/

## Supplementary Material

FIGURE S1 **(A)** Representative image of ZIKV-BR, ZIKV-AF, and MOCK infected neurospheres (NS). **(B)** Quantification of neurosphere size diameter in the MOCK, ZIKV-BR, and ZIKV-AF conditions. One-way ANOVA with Tukey’s multiple comparison test was used to identify the statistically significant comparisons. ^∗^Adjusted *p*-value ≤ than 0.05. ^∗∗∗^Adjusted *p*-value ≤ than 0.001. Ns, not significant with adjusted *p*-value > than 0.05.

FIGURE S2 Distribution of protein expression ratio according to protein function/localization.

FIGURE S3 **(A)** Representative image of neuronal enriched culture showing neurons in green and astrocytes in red. Nuclei stained in blue. **(B)** ZIKV-BR and MOCK infected neuronal culture detected by RT-qPCR. **(C)** Increase in the expression of Caspase 3 after ZIKV-BR infection of control-derived neurons was observed (*p* < 0.01).

TABLE S1 Neurospheres ZIKV-AF vs. ZIKV-BR vs. MOCK ANOVA regulated proteins FDR < 5%.

TABLE S2 Neurospheres ZIKV-BR vs. MOCK human Uniprot database *t*-test with Benjamini–Hochberg correction regulated proteins ZIKV Uniprot database. FDR < 5%.

TABLE S3 Neurospheres ZBvsMOCK_GO – Upregulated.

TABLE S4 Neurospheres ZBvsMOCK_GO – Downregulated.

TABLE S5 Neurospheres ZIKV-AF vs. ZIKV-BR vs. MOCK human mycrocephaly related genes Uniprot database ANOVA Regulated proteins FDR < 5%.

TABLE S6 NEURONS ZIKV-BR vs. MOCK human uniprot database *t*-test with Benjamini–Hochberg correction regulated proteins ZIKV Uniprot database FDR < 5% B-H FDR < 5%.

TABLE S7 Neurons ZIKV-BR vs. MOCK_GO – Upregulated.

TABLE S8 Neurons ZIKV-BR vs. MOCK_GO – Downregulated.

TABLE S9 Neurons PD7 ZIKV-BR vs. PD7 MOCK regulated synaptic protein families based on data mining.

TABLE S10 NPCs differenciation.

TABLE S11 List of peptides belonging to DCX, HSPB1, and ACTB monitored by PRM.

TABLE S12 List of antibodies used for validation of proteomics data.
