# Supplementary figures and images for: Zika Virus Impairs Neurogenesis and Synaptogenesis Pathways in Human Neural Stem Cells and Neurons
Source: Front Cell Neurosci. 2019 Mar 15;13:64. doi: 10.3389/fncel.2019.00064 (PMC6436085; doi:10.3389/fncel.2019.00064)

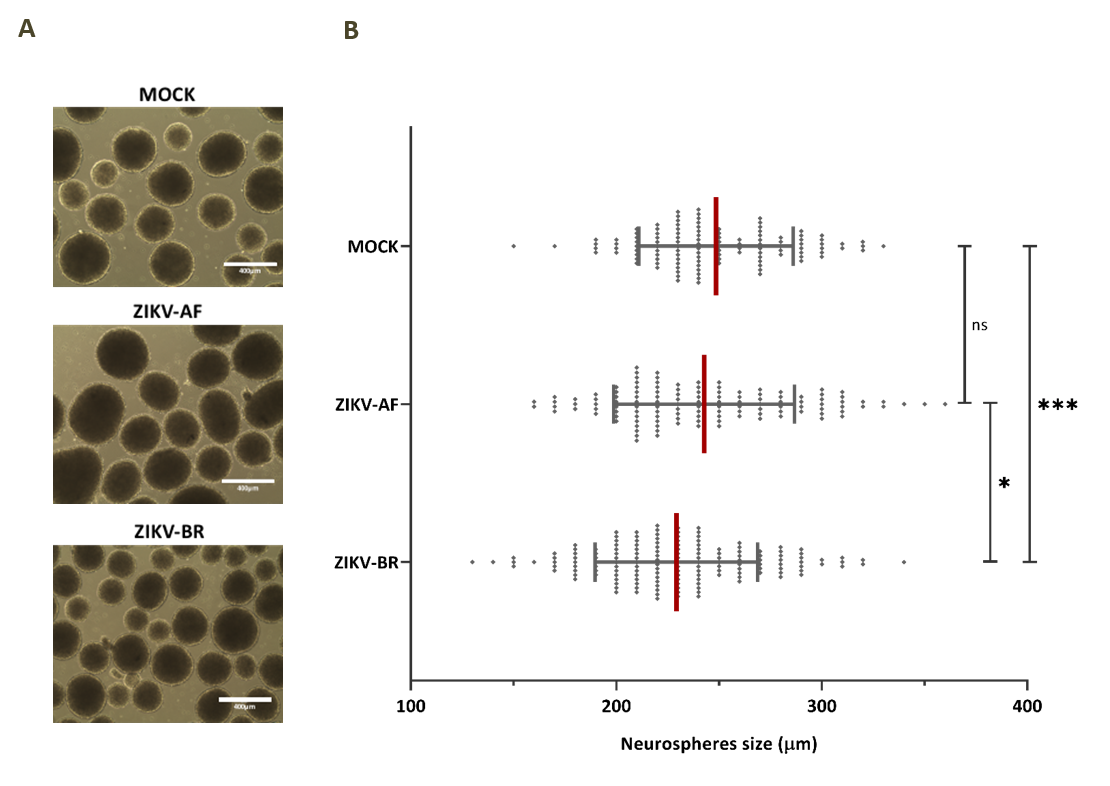

Supplement: FIGURE S1 — (A) Representative image of ZIKV-BR, ZIKV-AF, and MOCK infected neurospheres (NS). (B) Quantification of neurosphere size diameter in the MOCK, ZIKV-BR, and ZIKV-AF conditions. One-way ANOVA with Tukey’s multiple comparison test was used to identify the statistically significant comparisons. ∗Adjusted p-value ≤ than 0.05. ∗∗∗Adjusted p-value ≤ than 0.001. Ns, not significant with adjusted p-value > than 0.05. [file Image_1.TIF]

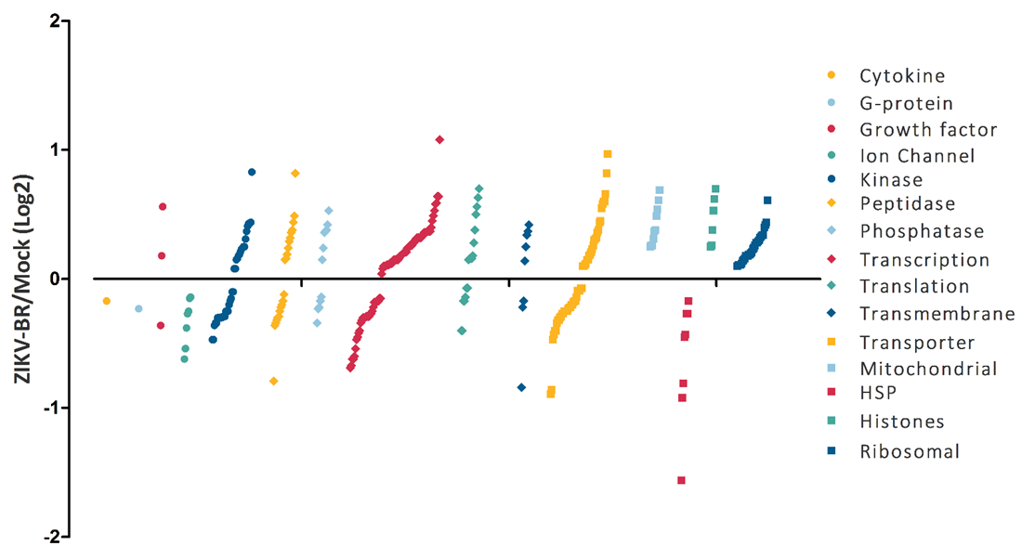

Supplement: FIGURE S2 — Distribution of protein expression ratio according to protein function/localization. [file Image_2.TIF]

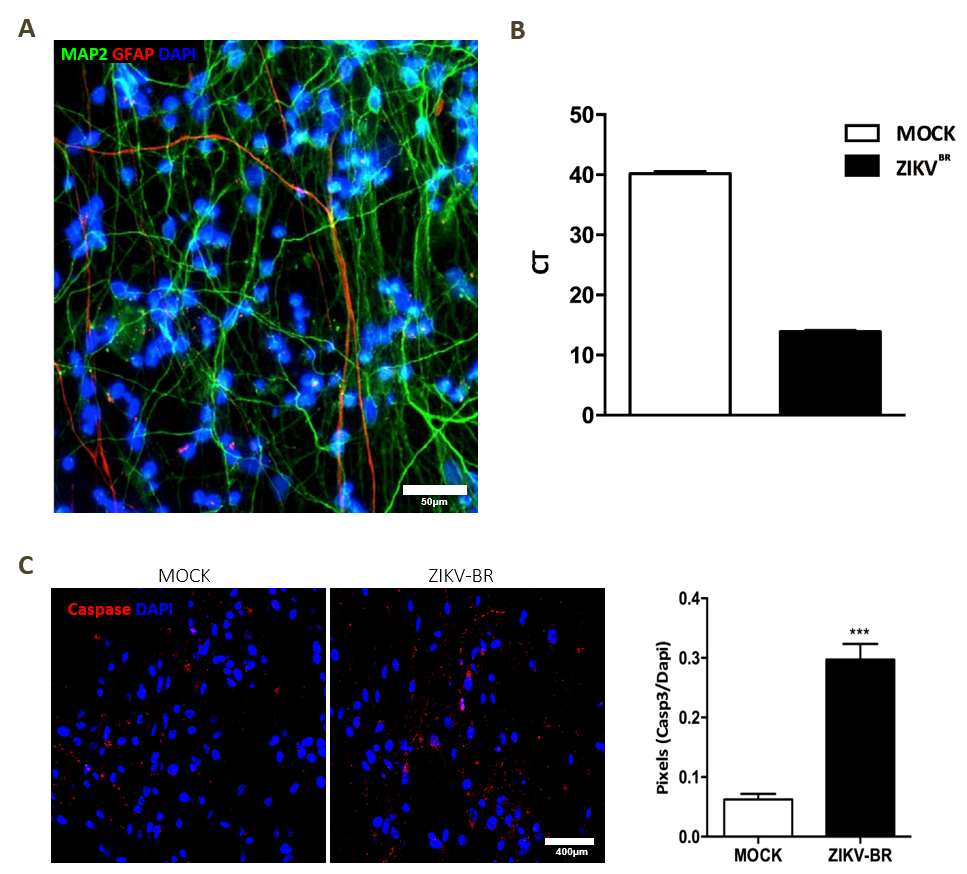

Supplement: FIGURE S3 — (A) Representative image of neuronal enriched culture showing neurons in green and astrocytes in red. Nuclei stained in blue. (B) ZIKV-BR and MOCK infected neuronal culture detected by RT-qPCR. (C) Increase in the expression of Caspase 3 after ZIKV-BR infection of control-derived neurons was observed (p < 0.01). [file Image_3.TIF]
